# Passive Immunization in JNPL3 Transgenic Mice Using an Array of Phospho-Tau Specific Antibodies

**DOI:** 10.1371/journal.pone.0135774

**Published:** 2015-08-13

**Authors:** Cristina d’Abramo, Christopher M. Acker, Heidy Jimenez, Peter Davies

**Affiliations:** Litwin-Zucker Center for Research in Alzheimer's Disease, Feinstein Institute for Medical Research, North Shore/LIJ Health System, Manhasset, NY, 11030, United States of America; University of Florida, UNITED STATES

## Abstract

Recent work from our lab and few others have strongly suggested that immunotherapy could be an effective means of preventing the development of tau accumulation in JNPL3 transgenic mice, carrying the human P301L mutation. The aim of this study was to test the efficacy of a variety of specific tau monoclonal antibodies in JNPL3. Starting at 3 months of age, mice were treated for 4 months with weekly intraperitoneal injections of saline or purified tau monoclonal antibodies (10mg/Kg) different in specificity for pathological tau: CP13 (pSer202), RZ3 (pThr231) and PG5 (pSer409). As expected, not all the antibodies tested showed efficacy at preventing the development of tau pathology at the described dose, with some of them even worsening the pathological scenario. Only by targeting the pSer202 epitope with CP13 was a conspicuous reduction of insoluble or soluble tau in cortex and hindbrain obtained. Here we report about the importance of screening in vivo multiple tau antibodies in order to select the antibodies to direct into future clinical studies.

## Introduction

Alzheimer’s disease accounts for 50–80% of senile dementias. In AD, neurofibrillary tangles (NFT) together with amyloid plaques and neuronal loss characterize brain pathology. The link between the two lesions is still unknown but the severity of the cognitive deficit appears to closely correlate with the amount and extent of NFT pathology. Tau pathology progresses in a predictable manner, going from one brain region to another according to a disease-specific pattern [1–5]. The “conventional” evolution pattern of tau lesions has been described with abnormal and hyperphosphorylated tau initially appearing in a non-fibrillar and non-argyrophilic form that is soluble to some degree in the neuronal cytoplasm and is referred to as “pretangle” material. Pretangles aggregate slowly, then undergo conversion into a β-pleated sheet conformation and finally convert into insoluble fibrils: NFT. Neurofibrillary changes of the Alzheimer type are resistant to autophagy and other endogenous cellular removal mechanisms [6–8]. However, recent findings have shown that “extracellular tau” might be the culprit in propagation of tau pathology as disease progresses. *In vitro* and *in vivo* experiments have demonstrated that aggregated tau can spread from cell to cell [9–14]. Nonetheless, the molecular nature of the extracellular propagating species is unknown. Many studies have suggested that an immunotherapy approach targeting tau pathology can effectively prevent or possibly block the progression of tau pathology in transgenic mouse models [15–24]. If it is assumed that antibodies are effective by blocking extracellular propagation of pathology, the nature of the epitopes recognized might provide information on the molecular nature of this species of tau.

The intent of this study was to test the efficacy of distinct p-tau monoclonal antibodies in prevention of the development of tau pathology in tau transgenic mouse models, and the variety of antibodies produced in our lab represents a useful platform for this purpose. The monoclonal antibodies used include two different p-tau Abs targeting phosphorylations appearing on both normal adult brain and PHF-tau, plus a phospho-Ab directed to a phosphorylation site found just on PHF-tau: CP13 (pSer202), RZ3 (pThr231) and PG5 (pSer409) [25–27]. As expected not all the antibodies exhibited the ability of reducing tau pathology. In fact, only CP13 was able to lower tau accumulation and phosphorylation in cortex and hindbrain.

## Materials and Methods

### Transgenic animals

JNPL3 homozygous transgenic animals were purchased from Taconic. Cohorts of female JNPL3 were used as a model of hyperphosphorylation and aggregation of tau protein. This transgenic line expresses human MAPT (4R0N) with the P301L mutation driven by the mouse prion promoter and develops neurofibrillary tangles in an age and gene–dose dependent manner, as early as 4.5 months. Starting at 3 months of age, mice were treated for 4 months with weekly intraperitoneal injections of purified tau monoclonal antibodies or saline. The antibodies were used at a dose of 10mg/Kg, and 1X phosphate buffered saline was used as a negative control. The Abs treated groups included 16 animals, the control group 29 mice, while the baseline cohort consisted of 22 mice sacrificed at 3 months of age. Because mice were only studied from ages 3 to 7 months, no effects on survival, deterioration of motor function or other phenotypes were expected or observed. Mice were individually handled and examined at least once a week during treatment. Euthanasia of mice was performed by decapitation under deep isoflurane anesthesia. Approval for the work reported here was obtained from the Feinstein Institute IACUC under protocol numbers 2007–029 and 2015–018.

### Tau monoclonal antibodies

All monoclonal antibodies used were produced in our laboratory and have been divided into different groups, based on specificity for pathological tau: two phospho-Abs targeting phospho-epitopes present on both normal adult brain and PHF-tau, CP13 (pSer202), RZ3 (pThr231); a phospho-Ab directed to a phosphorylation site found just on PHF-tau, PG5 (pSer409), The antibodies used in the study are IgG1, except for PG5 which is an IgG3. All three antibodies have serum half-lives of more than 2 weeks in P301L mice (unpublished data).

### Brain sample preparation

Upon sacrifice, brains were removed and processed as previously described [17, 25, 27]. Briefly, half of the brain was dissected for biochemical analysis: forebrain, hindbrain and hippocampus were homogenized separately using an appropriate volume of homogenizing buffer, a solution of Tris-buffered saline (TBS), pH 7.4, containing 10mM NaF, 1 mM Na_3_VO_4_ and 2mM EGTA, plus a complete Mini protease inihibitor cocktail (Roche). Homogenized brain samples were stored at -80C and used for separate measurements of soluble and insoluble tau. To examine the levels of soluble tau in the mouse samples, heat stable preparations were made by adding 5% 5M NaCl and 4% β-mercaptoethanol. Samples were then heated at 100°C for 10 minutes, cooled at 4°C for 15 minutes, and spun at 14,000 g for 10 min. This procedure effectively removes any mouse IgG (endogenous or exogenous) and simplifies the interpretation of the biochemical results [28–31]. To obtain the insoluble tau preparation [32, 33] 500μl of homogenate were thawed and spun at 6000g for 10’ at 4°C. The collected supernatant was centrifuged at 200,000g for 30’ at 25°C. Pellet was resuspended in 450μl of homogenizing buffer and spun again at 200,000g for 30’ at 25°C. Final pellet was resuspended in 200μl of 1X sample buffer.

### Low–Tau sandwich ELISA

Low-Tau sandwich ELISA was performed as already published [25]. 96-well plates (Nunc) were respectively coated with DA31, PHF1, CP13 or RZ3 at a final concentration of 6μg/ml in coating buffer, for at least 48h at 4°C. After washing 3X in wash buffer, the plates were blocked for 1h at room temperature using StartingBlock Blocking buffer (Thermo Scientific) to avoid non-specific binding. Each plate was then washed 5X and 50μl of the appropriate sample was added to the wells, with 50μl of DA9-HRP detection antibody. Plates were incubated O/N shaking at 4°C and then washed 9X in wash buffer. 1-Step ULTRA TMB-ELISA (Thermo Scientific) was added for 30’ at room temperature before stopping the reaction with 2M H_2_SO_4_. Plates were read with an Infinite m200 plate reader (Tecan) at 450nm.

### Tau Monoantibody ELISA

Tau Monoantibody sandwich ELISA was performed as already published [32, 33] in order to detect tau levels in the insoluble fraction of the hippocampus. Plates were coated with DA9 (total tau, AA 102–140) at a concentration of 6μg/ml in coating buffer for at least 48 hours at 4°C. Plates were washed 3x in wash buffer then blocked with StartingBlock (Thermo Scientific). 50ul of total lysates and PHF-tau, as a standard, were incubated overnight at 4°C. After washing 5x, the total tau detection antibody DA9-HRP was added and incubated for 2 hours at room temperature. The steps that follow are same as above.

### Immunocytochemistry

After fixing half brain in 4% paraformaldehyde overnight at 4°C, serial sections were cut on a vibratome and conserved in TBS (50mM Tris, 150mM NaCl, pH 7.6, 0.02% NaN_3_). The staining was done in multiwell plates using a standardized protocol [17]. Tau antibody CP13, diluted 1/5000 in 5% milk, was incubated overnight with shaking, followed by biotin-conjugated secondary IgG1 antibody diluted 1/1000 in 20% Superblock and Streptavidin-HRP. Staining was visualized by 3,3′-Diaminobenzidine. Each step was followed by 5x washes in TBS.

### Statistical analysis

Statistical analyses were performed with dedicated software (GraphPad Prism vs6). Data were analyzed by Unpaired t-test with Welch’s correction, with significance level set at p<0.05. Linear regression analysis was performed for all correlations.

## Results

### CP13 significantly reduces tau and p-tau in both cortex and hindbrain insoluble fractions

Starting at 3 months of age, JNPL3 mice received weekly intraperitoneal injections of either saline or a specific tau monoclonal antibody (10 mg/Kg). Animals received a total of 16 doses and were killed at 29 weeks. The antibody treatment was well tolerated with just one mouse death in the RZ3 group and 2 deaths in the PBS cohort.

**Fig 1:**
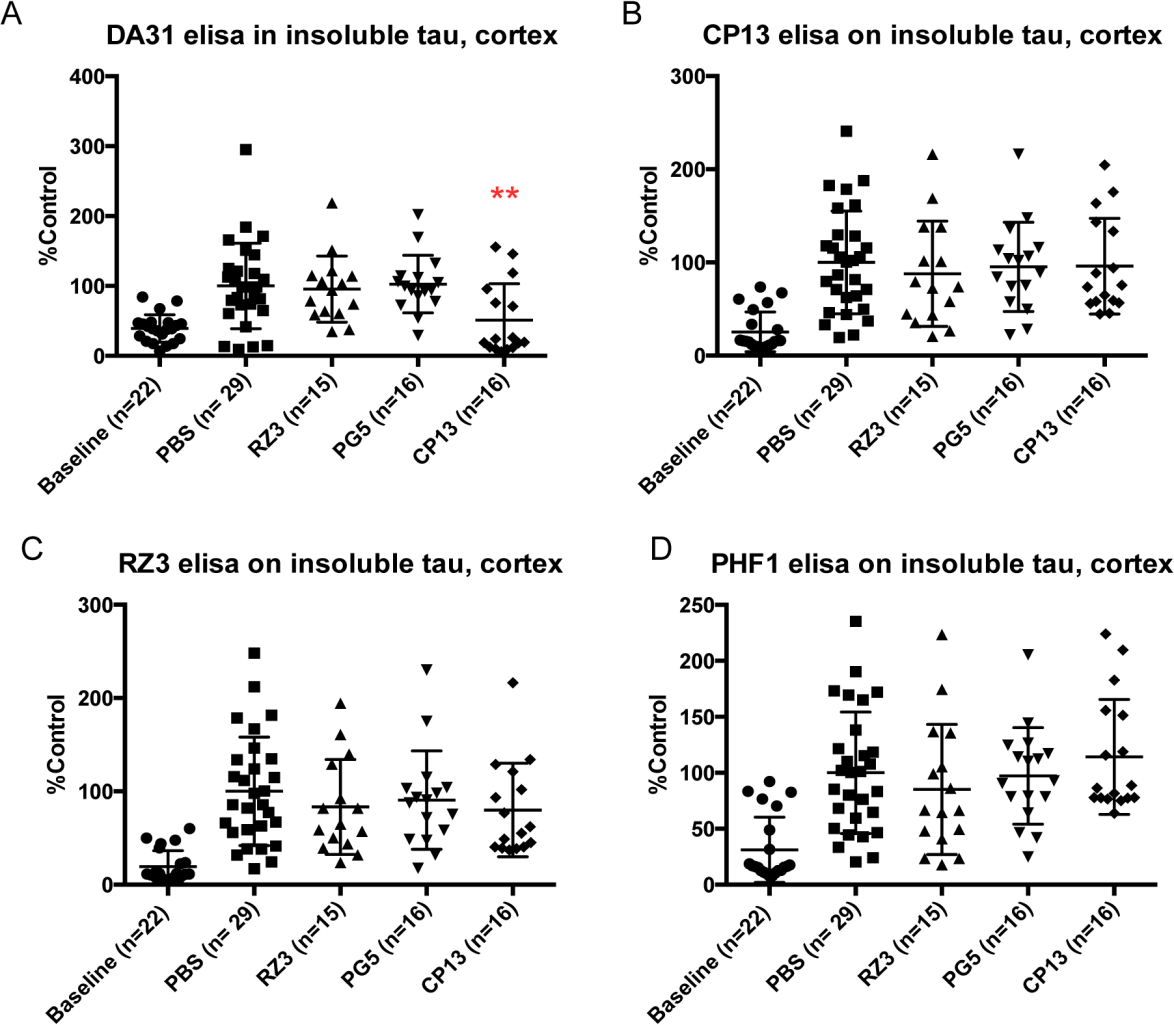
Effect of immunotherapy on insoluble tau in cortex. Mice were treated with different antibodies from 3 to 7 months of age. Baseline (3 months) and PBS cohorts were included in the study. (A) After 4 months of treatment CP13 significantly decreases insoluble tau in cortex (**p = 0.0073). (B-D) None of the other antibodies exert any effect on insoluble tau in cortex. Results are expressed as % PBS control.

**Fig 2:**
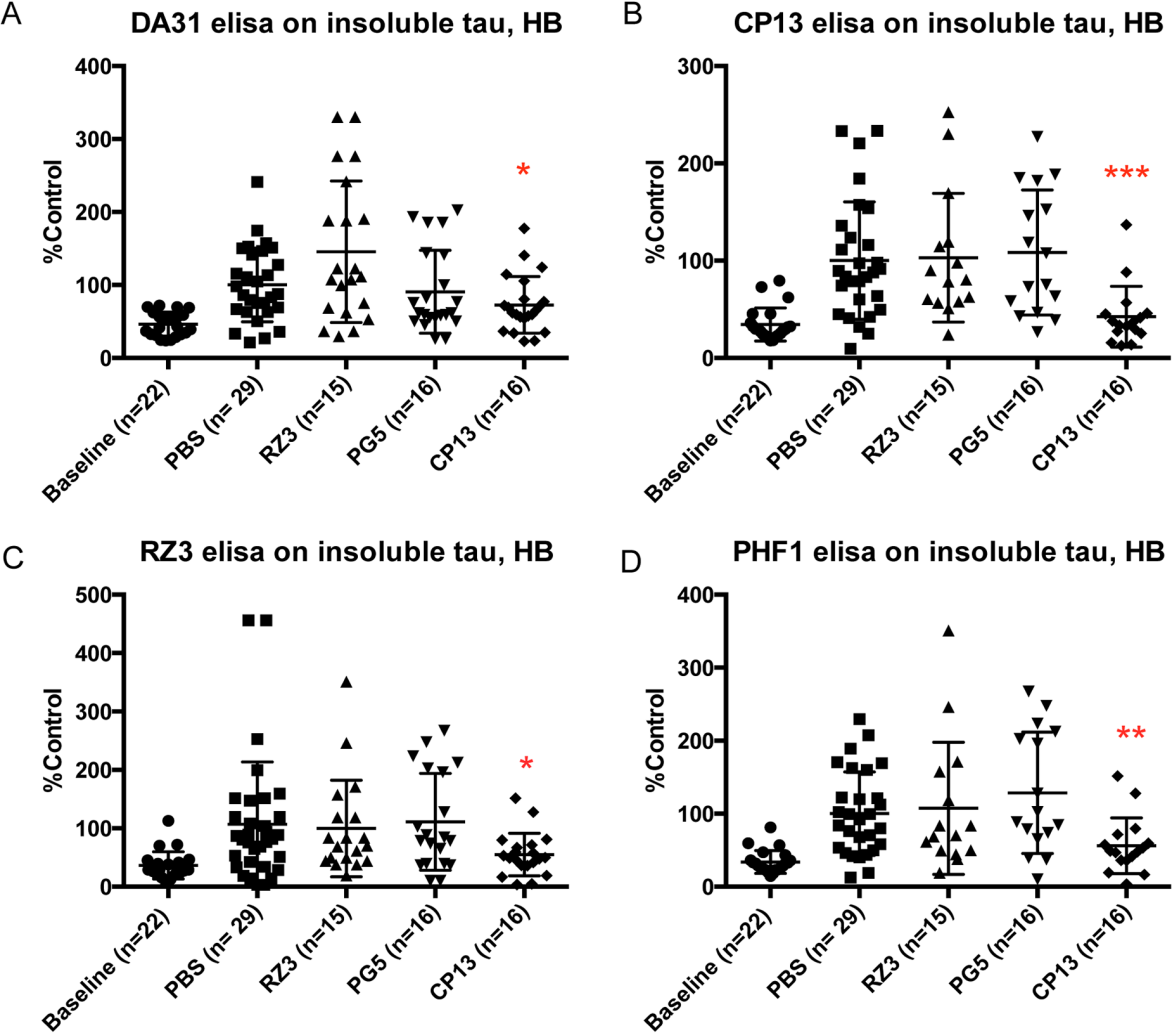
Effect of immunotherapy on insoluble tau in hindbrain. (A) After 4 months of treatment no effect is evident on total insoluble tau, while (B) CP13 significantly decreases insoluble pSer202 tau in hindbrain (***p = 0.0001), together with (C) pThr231 (*p = 0.0258). Results are expressed as % PBS control.

Analysis of the insoluble fractions revealed that CP13 was the only antibody able to effectively decrease tau pathology. In fact, the level of tau in cortex appeared to be significantly reduced (**p = 0.0073) compared to the control animals when targeting pSer202 with CP13 (Fig 1A). In parallel, insoluble tau in the hindbrain was decreased in terms of phosphorylation amount (Fig 2B and 2C): pSer-202 was significantly lowered (***p = 0.0001) together with pThr231 (*p = 0.0258) when treating mice with CP13.

Notably, none of the other antibodies selected in this immunotherapy study exerted any effect on insoluble cortex and hindbrain fractions (Figs 1 and 2).

### CP13 decreases tau soluble levels in hindbrain, with RZ3 triggering tau phosphorylation in the same fraction

**Fig 3:**
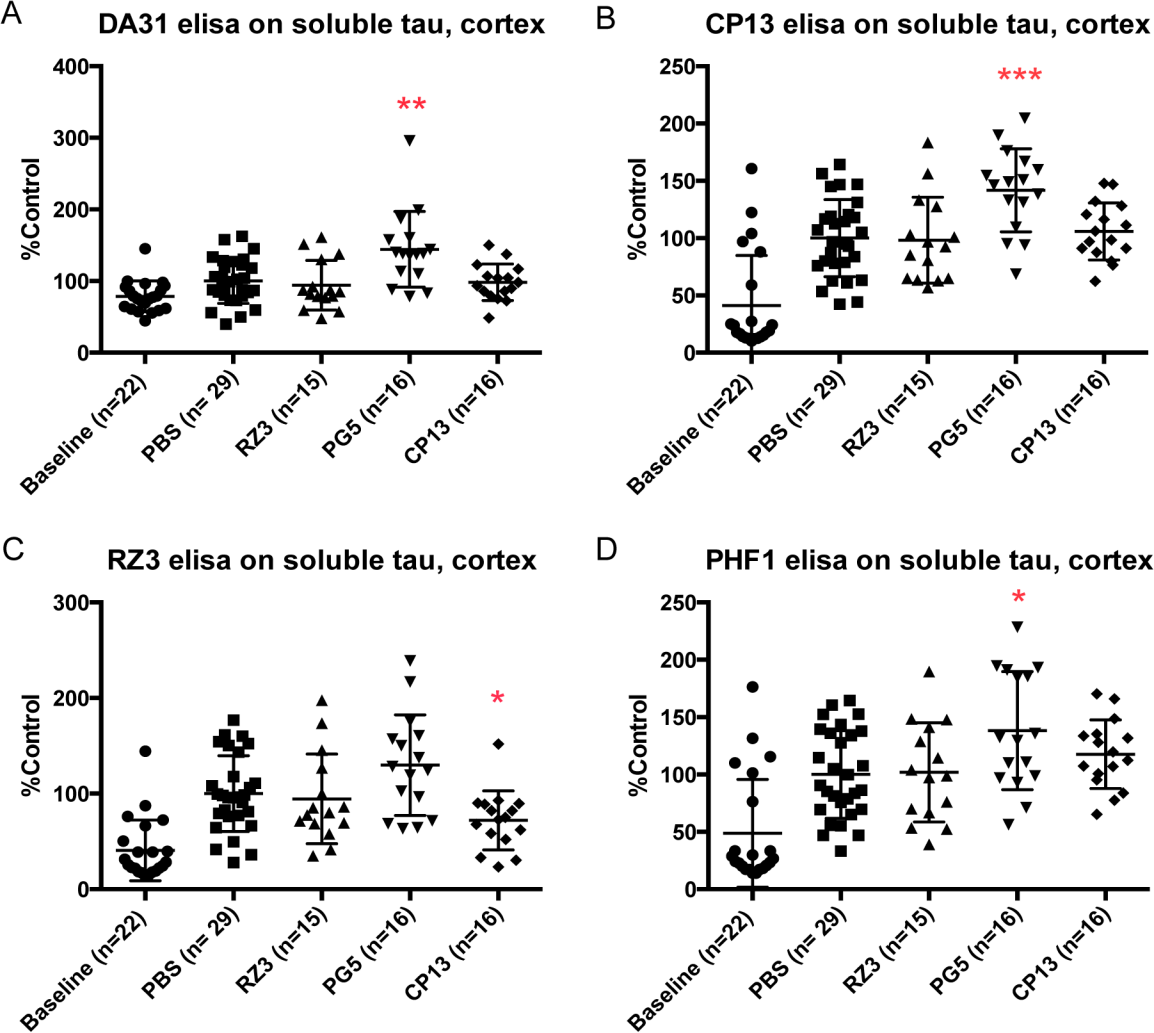
Effect of immunotherapy on soluble tau in cortex. (A) PG5 increases the accumulation of soluble tau (**p = 0.0058) in cortex. (B,D) Phosphorylations at Ser202 and Ser396-404 are significantly higher than the PBS group when injecting PG5 (***p = 0.0007 and *p = 0.0155 respectively). (C) RZ3 ELISA detecting pThr231 shows lower phosphorylation when treating animals with CP13 (*p = 0.0124)**.** Results are expressed as % PBS control.

**Fig 4:**
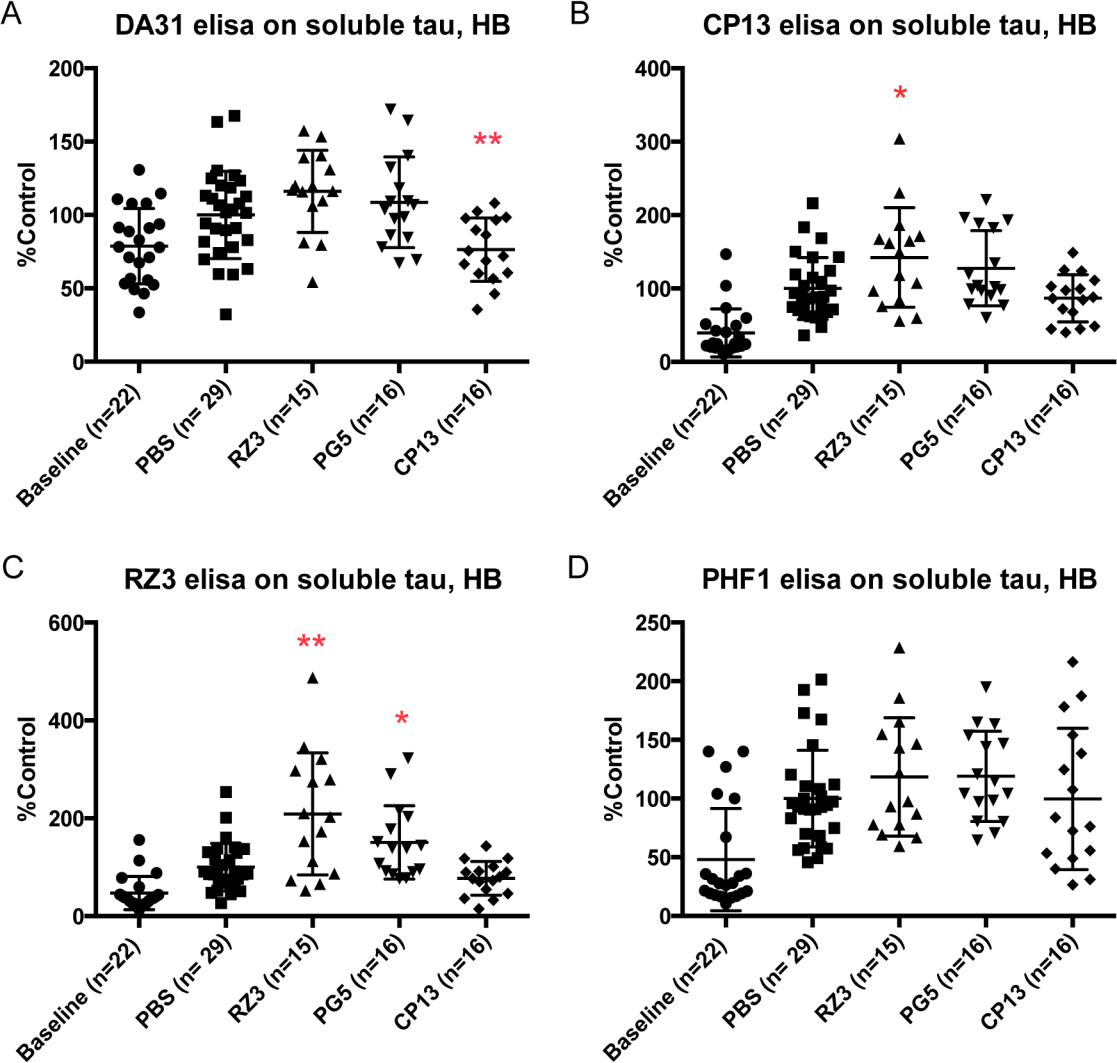
Effect of immunotherapy on soluble tau in hindbrain. (A) animals injected with CP13 show decreased soluble tau levels in hindbrain (**p = 0.0039). (B,C) RZ3 increases phosphorylation at both Ser202 (*p = 0.0394) and Ser231 (**p = 0.0049), while PG5 significantly augments pThr231 (*p = 0.0236). No effect has been reported on Ser396-404 (D). Results are expressed as % PBS control.

Biochemical investigation on soluble tau fractions revealed that CP13 lowered p-tau at Thr231 in cortex (*p = 0.0124), with significant reduction of total soluble fraction in the hindbrain region (**p = 0.0039) (Figs 3C and 4A). Noteworthy, RZ3 displayed increased levels of soluble phosphorylated tau in hindbrain, at Ser202 (*p = 0.0394) and Thr231 (**p = 0.0049) respectively (Fig 4B and 4C).

### PG5 worsens the pathological scenario in the soluble cortex fraction, with inconsistent effects in the hippocampus

PG5 caused a general remarkable accumulation of soluble tau levels (**p = 0.0058), pSer202 (***p = 0.0007) and pSer396-404 (*p = 0.0155) in cortex (Fig 3A, 3B and 3D), together with increased pThr231 (p = 0.0236) in soluble tau hindbrain.

**Fig 5:**
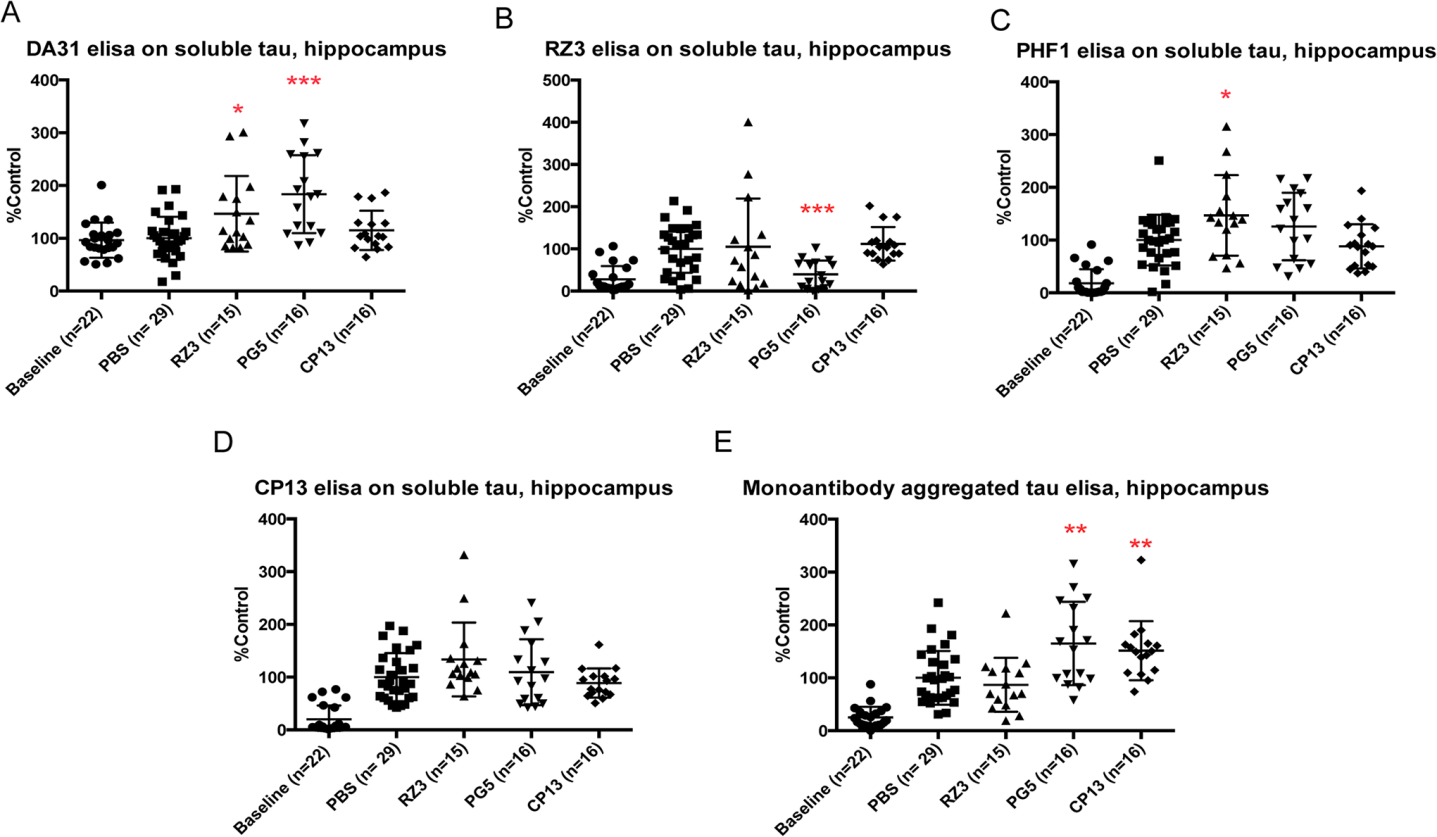
Effect of immunotherapy on soluble and on aggregated tau in hippocampal fractions. (A) PG5 and RZ3 increase soluble tau levels (***p = 0.0004 and p = 0.0317 respectively). (B) Phosphorylation at Thr231 on soluble tau is lowered by PG5 (***p<0.0001), while (C) pSer396-404 is increased when injecting RZ3 (*p = 0.0427). (E) The monoantibody ELISA to detect aggregated tau in the hippocampal fraction reveals increased levels of aggregated tau after injecting PG5 (**p = 0.0069) or CP13 (**p = 0.0049). Results are expressed as % PBS control.

Analysis of the hippocampal soluble fraction showed a boost of the tau burden (***p = 0.0004) with unexpected decreased phosphorylation at Thr231 (***p<0.0001) The mono-antibody aggregated assay on hippocampal lysates revealed a significant increase in insoluble tau when injecting the animal with PG5 (**p = 0.0069) (Fig 5A, 5B and 5E).

### RZ3 and CP13 increase aggregation of tau in hippocampal fractions

Besides increasing p-tau in soluble hindbrain samples, RZ3 significantly incremented soluble tau (*p = 0.0317) and pSer396-404 (*p = 0.0427) in the hippocampal region (Fig 5A and 5C). Inconsistent with the data above, CP13 augmented tau aggregation in the hippocampus (**p = 0.0049) (Fig 5E).

### Immunocytochemistry confirms CP13 and PG5 effects.

**Fig 6:**
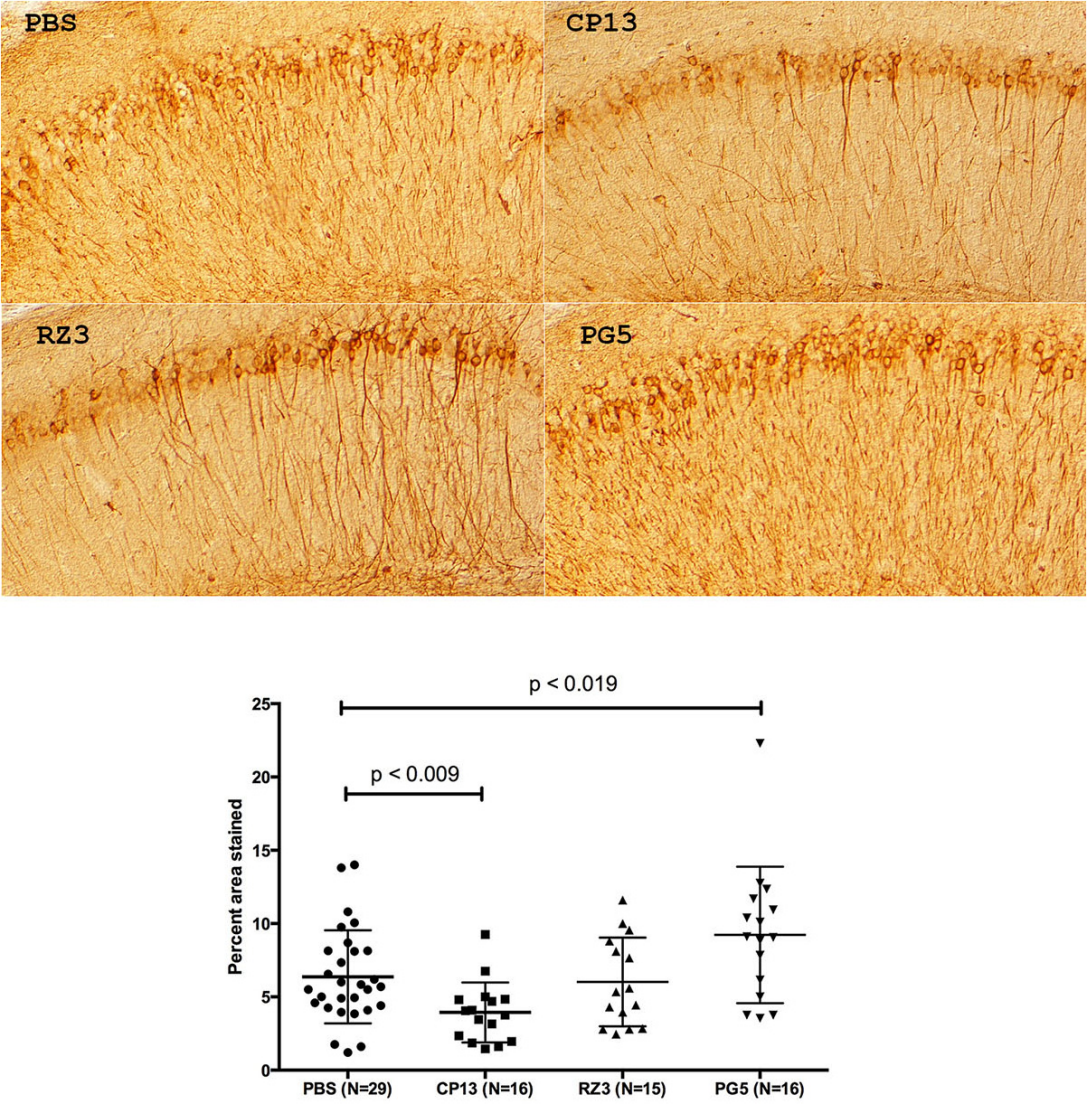
CP13 immunocytochemistry. Representative stained sections from each treatment group are shown. Images as shown were analyzed with ImageJ for percent area stained. Each data point represents the mean value obtained from at least two sections per mouse. The area stained is significantly lower from CP13 treated mice (p < 0.009) and significantly higher in PG5 treated mice (p < 0.019).

Immunohistochemistry was performed using CP13 as primary antibody. Overall, no qualitative changes in tau accumulation were obvious following treatment with any of the antibodies. Quantitative studies were only performed in the hippocampus, where the area stained by CP13 was measured in the CA1 region. Examples of staining are shown in Fig 6. The area stained was significantly lower in CP13 treated mice (**p<0.009), and significantly higher in PG5 treated animals (*p<0.019) (Fig 6). Note that reduced neuronal staining is unlikely to be due to masking of epitopes in neurons: we have been unable to detect any of the monoclonal antibodies within neurons following the treatment schedule used [17]

## Discussion

A good amount of literature was published in the past five years on tau immunotherapy. Among that, targeting p-tau epitopes seemed the most straightforward strategy to apply since tau is hyperphosphorylated within the NFTs. Passive or active immunotherapy against pSer396-404, pSer422 and pSer231 were shown to reduce tau pathology at different degrees and are currently in clinical development [16, 21, 24, 34].

The purpose of this study was to test the efficacy of additional specific p-tau monoclonal antibodies in order to prevent/clear tau pathology in transgenic mouse models of AD and tauopathies.

JNPL3 animals, carrying the tau mutation responsible for frontotemporal dementia in humans, were treated with weekly intraperitoneal injections of distinct p-tau antibodies: CP13 (pSer202), RZ3 (pThr231) and PG5 (pSer409). A mock-treated cohort of mice was injected with PBS, and a group of animals was sacrificed at 3 months of age in order to have a baseline to refer to. Brains were dissected into cortex, hindbrain and hippocampus in order not to miss any biochemical effect due to potential “dilution” of pathology within the diverse areas. Biochemical analysis of cortex, hindbrain and hippocampus showed increased levels of insoluble tau in the PBS group compared to the baseline cohort, as a confirmation of the aging/pathological process in P301L mice going from 3 to 7 months of age.

As expected, antibodies treatments affected tau levels and phosphorylation in different ways, with CP13 being the only one really ameliorating the pathological scenario. CP13 recognizes a phospho-tau epitope present on both normal and pathological tau, with hyperphosphorylation at Ser202 occurring early in the pathology. In our study, CP13 significantly decreased insoluble tau levels both in cortex and hindbrain, with a lower phosphorylation pattern (pSer202, pSer231) described in the hindbrain insoluble fraction. Moreover CP13 acted reducing soluble tau in the hindbrain region, with diminished pThr231 soluble tau in cortex. An inconsistent effect was reported in terms of aggregation in the hippocampus, with increased amount of reactivity in the tau monoelisa. This assay captures aggregates as small as dimers, meaning that what we might encounter here is an accumulation of smaller aggregates while the amount of the larger (pelleted) aggregates decreases.

RZ3 is a monoclonal antibody targeting Thr231 and belonging to the same group of Abs as CP13. When targeting Thr231, JNPL3 displayed increased levels of soluble pSer202 and pThr231 in hindbrain, plus higher levels of total soluble tau in the hippocampus. This result means that even though RZ3 and CP13 target similar epitopes the effect on tau pathology can be very different. Moreover, a recent study [21] using higher concentration of an Ab targeting the Thr231 epitope has shown reduced AT8-immunoreactivity p-tau in the hippocampal fraction of the tg4510 animals. The inconsistency of these data compared to ours probably resides in the different Ab concentration used, together with divergent mouse models and brain areas analyzed.

Continuing in the interpretation of our data, PG5 has shown questionable effects. This immunoglobulin targeting pSer409 is part of a group of antibodies reactive to phosphorylation sites found just on PHF-tau, and we assumed that this Ab would have been more “pathology-specific”. Surprisingly, PG5 worsened total tau accumulation and boosted phosphorylation at Ser202 in the cortex soluble fraction, with increased pThr231 in hindbrain soluble tau. Hippocampal soluble and aggregated tau levels were enhanced by treating mice with PG5, while pThr231 resulted in significant reduction. Dissecting the brain in specific areas shows how Abs can act differently according to the area considered. This suggests how critical it is to focus on different regions in order to get information related to how and where the Ab might work.

Overall, in the present study CP13 was the only Ab showing a real efficacy in preventing the development of tau pathology essentially allowing baseline levels to be maintained, although, with a different degree of success according to the brain areas and tau fractions considered.

While some generalizations may now be possible about which tau epitopes to target, there is a real need to examine multiple different mouse models and examine dose-response relationships for antibodies that appear efficacious. Furthermore, future experiments using tau antibodies will shed light on the nature of the tau species targeted during the study.

## Supporting Information

S1 File The GraphPad Prism file contains all the individual mouse ELISA data used in this manuscript, as well as the quantitative data from the staining of sections of hippocampus. The data files, individual graphs and composite figures are all included.
(ZIP)
